# Tensor Hypercontraction Form of the Perturbative Triples Energy in Coupled-Cluster Theory

**DOI:** 10.1021/acs.jctc.2c00996

**Published:** 2023-02-17

**Authors:** Andy Jiang, Justin M. Turney, Henry F. Schaefer

**Affiliations:** † Center for Computational Quantum Chemistry, Department of Chemistry, University of Georgia, Athens, Georgia 30602, United States; ‡ Center for Computational Molecular Science and Technology, School of Chemistry and Biochemistry, School of Computational Science and Engineering, Georgia Institute of Technology, Atlanta, Georgia 30332-0400, United States

## Abstract

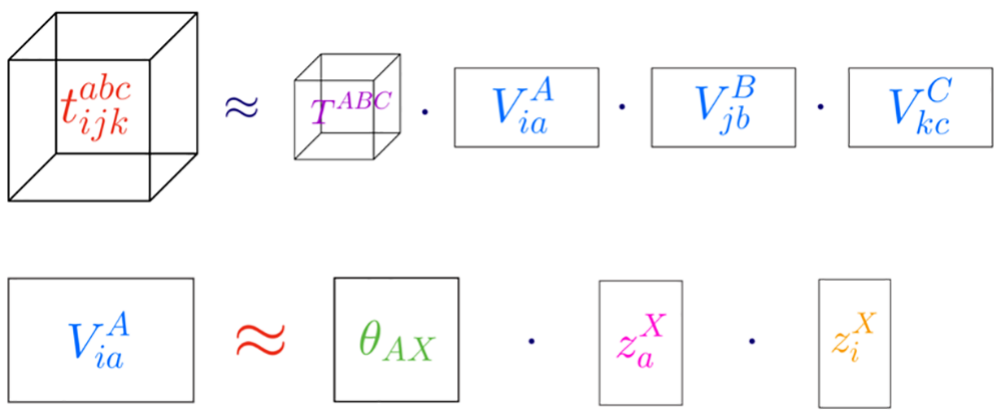

We present the working equations for a reduced-scaling method of evaluating the perturbative triples (T) energy in coupled-cluster theory, through the tensor hypercontraction (THC) of the triples amplitudes (*t*_*ijk*_^*abc*^). Through our method, we can reduce the scaling of the (T) energy from the traditional  to a more modest . We also discuss implementation details to aid future research, development, and software realization of this method. Additionally, we show that this method yields submillihartree (mEh) differences from CCSD(T) when evaluating absolute energies and sub-0.1 kcal/mol energy differences when evaluating relative energies. Finally, we demonstrate that this method converges to the true CCSD(T) energy through the systematic increasing of the rank or eigenvalue tolerance of the orthogonal projector, as well as exhibiting sublinear to linear error growth with respect to system size.

## 1 Introduction

Coupled-cluster (CC) theory^1,2^ is one of the most important advances of modern quantum chemistry, allowing for a polynomial-time evaluation of the electronic energies and wave function of a molecule, as a size-extensive alternative to truncated configuration interaction (CI) methods.^3,4^ Truncated CC methods also avoid the intractable superexponential scaling of full configuration interaction (FCI), yielding reasonable and chemically accurate relative energies compared to both the FCI limit and to experimental results, especially in the context of CCSD(T), also known as the “gold standard” method in computational quantum chemistry.^5^ The tractability and accuracy of CC methods make the development of efficient CC methods crucial for the future of quantum chemistry, as evaluation of accurate energies and wave functions is made possible for larger and more complex systems through hardware advances such as massively parallel computing^6−22^ and GPUs.^23−29^

However, there is still a tremendous gap in applicability between coupled-cluster theories (formally scaling at least ) and lower-scaling methods like Møller–Plesset perturbation theory (MP2)^30,31^ and density functional theory (DFT)^32,33^ (scaling  or better). Because of this, DFT and MP2 can be run on system tens or even hundreds of times the size of a system typically evaluated with CC methods.^34,35^ To close the gap between CC and less reliable electron correlation methods, it is useful to devise approximation schemes to CC which reduce the scaling, but also allow a means to systematically control the error compared to the nonapproximated CC method. One such approach involves local correlation,^36−47^ such as used in the DLPNO methods.^48,49^ With large enough molecules, these methods achieve asymptotic linear scaling.

Another approach is the rank reduction of the coupled-cluster amplitudes,^50^ using orthogonal projectors that transform the single and double cluster amplitudes into a smaller basis
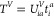
1
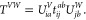
2Because of the orthogonal nature of the projectors, getting the full amplitudes from the rank-reduced form is trivial
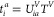
3
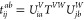
4

As shown by Parrish et al., the size of the *V* and *W* indices, also known as the projector rank, can be made directly proportional to the system size, while maintaining a set relative error from the absolute energy of a molecule.^50^ More recently, Hohenstein et al. have shown how to create a tensor hypercontracted (THC) form of the *t*_*ij*_^*ab*^ amplitudes, through the CANCENCOMP/PARAFAC (CP) decomposition^51^ of the orthogonal projectors.^52^
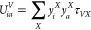
5
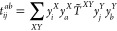
6
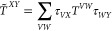
7Hohenstein et al. have also shown that, in the context of CCSD, the size of the *X* index can be made proportional to the system size to maintain a set relative error. Rank-reduction methods have also been applied to coupled-cluster theories involving higher levels of excitation, recently by Lesiuk with the SVD-CCSDT method,^53^ where the concept of orthogonal projectors is used to approximate the triples amplitude in CCSDT theory
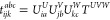
8

In the following sections, we combine the concepts of orthogonal projectors and THC to develop working equations for a reduced-scaling variant of the noniterative perturbative triples correction to the CCSD energy.^5^ Recently, Lesiuk derived an  approach to the (T) energy with orthogonal projectors which he calls RR-CCSD(T).^54^ In the current paper, we improve upon the work of Lesiuk’s approach utilizing tensor hypercontraction. Similar to how Hohenstein et al. used tensor hypercontraction to improve upon the RR-CCSD method of Parrish et al.,^50,52^ our method uses tensor hypercontraction to improve upon Lesiuk’s RR-CCSD(T) method.^54^ Our new approach, which we name THC-RR-CCSD(T), will commensurately enhance RR-CCSD(T), reducing the scaling of Lesiuk’s from  to . For consistency, we use many of the same formalisms as Lesiuk^53,54^ and Hohenstein et al.^52^

## 2 Theory

### 2.1 Notation

We use the following conventions to describe the indices appearing in this work:

- *i*, *j*, *k*, *l*: Occupied molecular orbitals, which range from 1 to *n*_*occ*_.
- *a*, *b*, *c*, *d*: Virtual molecular orbitals, which range from 1 to *n*_*virt*_.
- *P*, *Q*: Auxiliary indices of density-fitted/Cholesky-decomposed ERIs, which range from 1 to *n*_*aux*_.
- *w*, *v*: Laplace denominator weight indices, which range from 1 to *n*_*w*_.
- *U*, *V*, *W*: Rank-reduced dimensions of the doubles orthogonal projector, which range from 1 to *n*_*proj*_.
- *A*, *B*, *C*: Rank-reduced dimensions of the triples orthogonal projector, which range from 1 to *n*_*proj*_.
- *X*, *Y*, *Z*: CP-decomposition ranks of the triples orthogonal projector, which range from 1 to *n*_*proj*_.

The relative sizes of the indices are as follows:
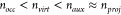
9Note that *n*_*w*_ does not grow with increasing molecular system size, and therefore, run-time analysis of intermediates with *w*, *v* indices will only treat the Laplace index as a prefactor.

The frozen-core approximation was used in all post-Hartree–Fock computations in this work; i.e., the 1*s* electrons are not correlated for all first-row atoms. The occupied space *n*_*occ*_ always refers to the number of correlated occupied orbitals. The generalized Einstein summation convention is used throughout—all indices appearing on the right-hand side but not on the left-hand side of an expression are summed over.

### 2.2 Perturbative Triples Correction to CCSD

CCSD is often not sufficient to obtain “chemically reliable” theoretical predictions, and it has been shown that only after triple excitations are considered that relative energies of under 1 kcal/mol can be regularly achieved.^55−61^ However, an explicit treatment of all triples has a very high cost of . Therefore, the triples amplitudes are often determined in a perturbative manner, based on the work of Raghavachari et al.^5^ In their formalism, the perturbative triples correction to the CCSD energy is defined as
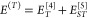
10where
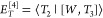
11
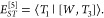
12*T*_1_, *T*_2_, and *T*_3_ are known as the “cluster operators” and, in second-quantization formalism, are defined as
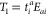
13
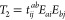
14
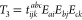
15*E*_*ai*_ represents the singlet, spin-adapted excitation operator, and is defined as
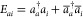
16where the barred creation/annihilation operators refer to the beta spin orbitals and nonbarred refer to the alpha spin orbitals.

The accuracy of the (T) method stems from a highly favorable error cancellation between *E*_*T*_^[4]^ and *E*_*ST*_^[5]^. In restricted, single-reference, closed-shell coupled cluster theory, one can write the equation for the (*T*) correction as^62^
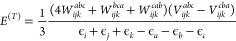
17where
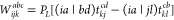
18and
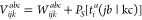
19Following the formalism of Lesiuk,^53^ we define *P*_*L*_ and *P*_*S*_, or the “long” and “short” permutation operations, as
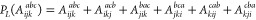
20
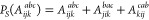
21

The perturbative triples amplitude (*t*_*ijk*_^*abc*^) is defined as
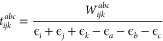
22

Using the perturbative triples amplitude, as well as the permutational symmetry of the Laplace denominator, one can rewrite eq 17 as
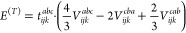
23

We use this equation when deriving the formulas for the THC-RR-CCSD(T) energy.

The cost of evaluating expression 23 scales as . However, the cost of evaluating expression 18 scales as , leading to an overall unfavorable  scaling of the CCSD(T) method.

### 2.3 Orthogonal Projectors

One crucial step of rank-reduced coupled cluster methods is the formation of the orthogonal projectors to reduce the dimensionality of the amplitudes, as given in eqs 1–4 and 8. There are a variety of methods that can be used to compute orthogonal projectors. One such method for the CCSD doubles amplitude is to form them from the definition of the MP2 *t*_*ij*_^*ab*^ amplitudes.^50^
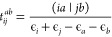
24Using density fitting (DF),^63,64^ also known as resolution-of-the-identity (RI), or Cholesky Decomposition (CD),^65^ the set of electron-repulsion integrals (ERIs) in the molecular orbital (MO) basis (*ia*|*jb*) can be written as follows:^66^
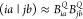
25

The energy denominator can be factored with a constant-sized index *w* (with growing molecular system size) through the Laplace denominator approach^67^
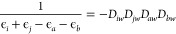
26

Combining these techniques, and the following intermediates, as defined by Parrish et al.,^50^
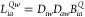
27
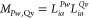
28allows us to diagonalize *M* and form the MP2 projector (*U*_*ia*_^*V*^) as
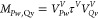
29
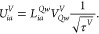
30Note that the size of the index V can be truncated based on the magnitude of the corresponding eigenvalue τ^*V*^. Even though the diagonalization of *M* is technically cubic scaling, the size of the *w* index can provide a large prefactor. In the case of larger molecules, the size of the *V* index is often much smaller than the size of the [*Qw*] index, and thus, truncated diagonalization approaches like the one given in ref (68) may be used. Overall, this approach scales . Similarly, projectors can be derived from MP3, albeit the equations are more complex,^50,54^

For triples amplitudes, we present two approaches devised by Lesiuk. In his SVD-CCSDT algorithm,^53^ he took guess *t*_*ijk*_^*abc*^ amplitudes, such as from CC3, and applied either a TUCKER-3 decomposition (scaling ) or an iterative SVD approach (scaling ), yielding the form of eq 8.

In his RR-CCSD(T) paper, Lesiuk devised an  scheme to compute projectors from the form of the perturbative triples amplitudes (eq 22), in a variant of HO-OI (Higher Order-Orthogonal Iteration).^54^ The steps of the algorithm are as follows:

- Start with the a guess of the triples projector *V*_*ia*_^*A*^. This can be done naively by setting *V*_*ia*_^*A*^ = *U*_*ia*_^*A*^ from the doubles amplitudes.
- Evaluate *t*_*ia,BC*_ from the current guess of the triples amplitudes, where
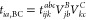
31By using the explicit expression for *t*_*ijk*_^*abc*^ and *W*_*ijk*_^*abc*^, this can be evaluated in . The working equations are presented in ref (54).
- Compute the SVD of *t*_*ia,BC*_ and take the largest *n*_*proj*_ left singular vectors as the next *V*_*ia*_^*A*^. This can be done in  time using a modified variant of truncated SVD, given in ref (68). In this algorithm, we save the singular values of this step (σ_*A*_), when we perform the CP decomposition of the triples projector. The pseudocode for this is presented in Section 4.
- Iterate until convergence. Convergence is defined when the difference between the Frobenius norm of the rank-reduced triples amplitudes *t*_*ABC*_, defined as
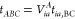
32between two successive iterations, falls below 10^–5^.

Since the source of the orthogonal projectors is not relevant to the scope of this paper, we only present results from computations utilizing the MP2 projector for the doubles amplitudes, and Lesiuk’s HO-OI approach for the perturbative triples amplitudes.

### 2.4 Tensor Hypercontraction (THC)

Tensor hypercontraction (THC) can be viewed as a “double approximation”, where two auxiliary indices are introduced to fit a high-dimensional tensor instead of just one. The THC form of electron repulsion integrals is defined as^69^
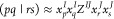
33This can be derived from the CP decomposition of *B*_*ia*_^*Q*^, (eq 25)
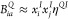
34
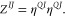
35Similarly, the THC form of coupled-cluster amplitudes can be derived from the tensor hypercontraction of the orthogonal projectors, given by, in the case of the doubles projector^52^
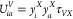
36

For the triples projector, it assumes a very similar form
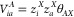
37

A PARAFAC/CANDENCOMP (CP) decomposition approach on *V*_*ia*_^*A*^ may be used. This approach is not dependent on the source of the projectors, and any of the projector building approaches from Section 2.3 may be used. Here, we use the variant of CP decomposition, first introduced by Hohenstein et al. for the doubles projector,^52^ where the eigenvalues of the doubles projector are in the CP decomposition, into the alternating least-squares (ALS) iterations.

In our algorithm, for the decomposition of the triples amplitude, instead of using the eigenvalues of the doubles projector, we use the singular values of the *t*_*ia,BC*_ intermediate (σ_*A*_). The functional to minimize is hence
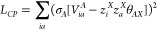
38

The update rule for each intermediate is given as
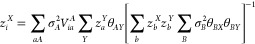
39
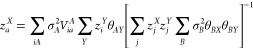
40
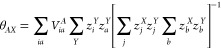
41

Note that the update rule for θ is the same as in traditional CP decomposition.

Since a CP decomposition does not exactly recreate the original projector, the projectors lose their orthogonal property.^52^ Therefore, we have to recreate the projectors after the CP decomposition
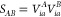
42
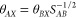
43
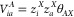
44

The *t*_*ijk*_^*abc*^ amplitudes can now be rewritten as, from eq 8
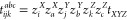
45
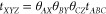
46

Recently, Hohenstein et al. have devised an algorithm that takes advantage of the THC form of the *t*_*ij*_^*ab*^ amplitudes to develop an  scaling implementation of CCSD.^52^ In the next section, we show how to extend this to the (T) correction with the THC form of the *t*_*ijk*_^*abc*^ amplitudes.

## 3 Derivation of Working Equations

We first define a couple of intermediates. From Lesiuk,^54^ we define
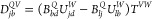
47

Next, we define the following chain of intermediates from contracting the polyadic vectors (*z*_*i*_^*X*^ and *z*_*a*_^*X*^) of the triples projector with the doubles projector, the DF/RI or CD decomposed ERIs, and the D intermediate from eq 47, as well as the *T*_1_ amplitudes.
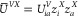
48
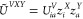
49
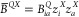
50
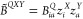
51
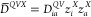
52
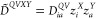
53
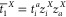
54
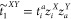
55

We then take eqs 23, 19, and 45, and the previously defined intermediates, to arrive at the THC form of the triples energy correction
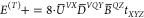
56
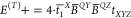
57
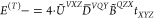
58
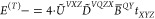
59
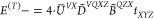
60
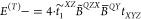
61
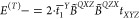
62
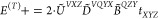
63
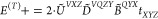
64
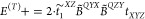
65

Equations 56 and 57 correspond to the first term in eq 23, eqs 58–62 the second term, and eqs 63–65 the third term. All of the contractions can be determined in  time or less.

## 4 Implementation Details

To aid future research and development, we present pseudocode for some of the algorithms we use for the optimal contraction of intermediate terms to evaluate the THC-RR-CCSD(T) energy. We first present our noniterative SVD algorithm to factorize the *t*_*ia,BC*_ intermediate, inspired by the truncated SVD and diagonalization algorithms given in ref (68). In Algorithm 1, we present a noniterative truncated SVD algorithm to avoid the *O*(*N*^6^) scaling of a traditional SVD of the *t*_*ia,BC*_ intermediate. In Algorithms 2–4, we present suggested contraction orders, as well as tensor slicings, for each term of the THC-RR-CCSD(T) energy expression. We try to make the contractions such that highly efficient level 3 BLAS matrix multiplication calls are utilized as much as possible. For each step of each algorithm, the runtime is given, and if a level 3 BLAS matrix multiplication call is possible, then the term (GEMM) is added. Additionally, the *D̃*^*QVXY*^ intermediate is never fully built to help with memory costs. The runtime of this algorithm is , with  storage costs; the only quartic memory requirements involve the storage of the *t*_*ia,BC*_ and *D*_*jb*_^*QV*^ intermediates. It may be possible to reduce the memory cost in future implementations of this method, but that is beyond the scope of this paper.

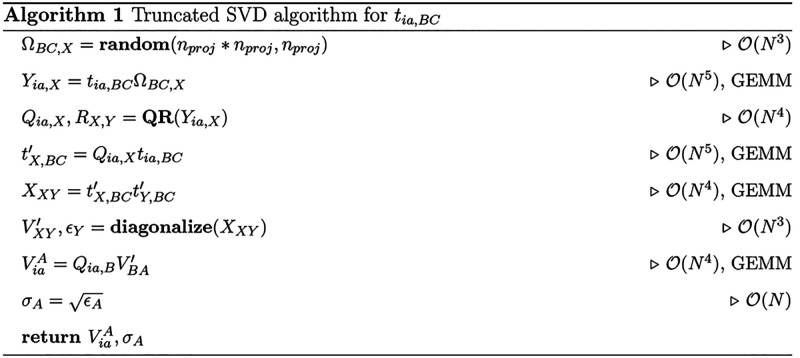

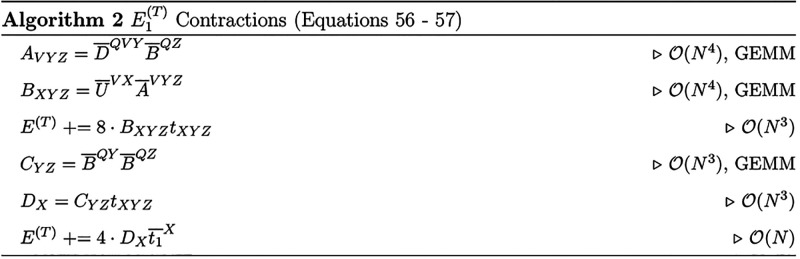

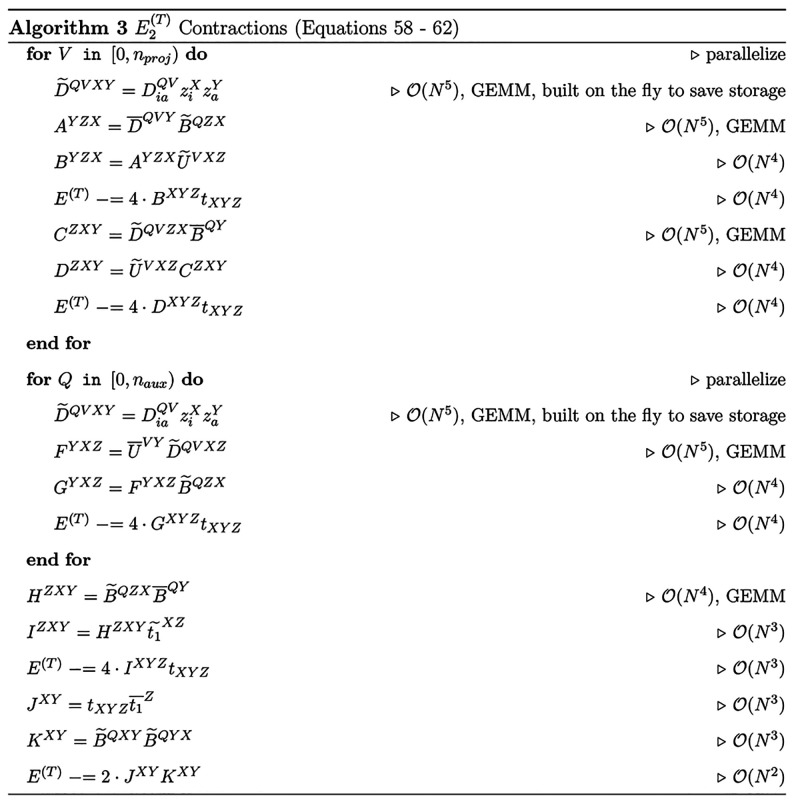

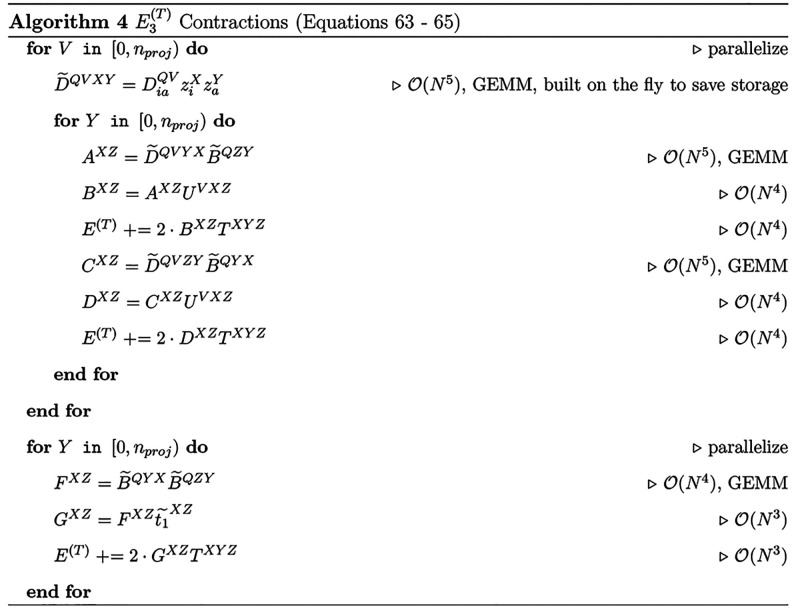









The code is implemented in a developmental plugin version of the Psi4 Quantum Chemistry code,^70^ following the completion of an exact CCSD computation. Tensor contractions are performed with the help of the EinsumsInCpp software (public on GitHub). The compressed doubles amplitudes *T*^*VW*^ used to build the triples projector are formed by transforming the exact CCSD amplitudes from the preceding computation by the MP2 projector amplitudes. This method is designed to be fully compatible and used with Hohenstein’s THC-RR-CCSD method.^52^ Future studies of using THC-RR-CCSD(T) in conjunction with THC-RR-CCSD are encouraged.

## 5 Results

### 5.1 Conformation Energies

We first evaluate our new THC-RR-CCSD(T) method on the CYCONF^71,72^ data set, a set containing 11 different conformations of gaseous cysteine, with 10 corresponding conformation energies, relative to the lowest conformer. We evaluate conformation energies for each of the 10 conformations in CCSD, CCSD(T), and THC-RR-CCSD(T), and for each system, and we use the exact CCSD(T) conformation energy as the reference. We do this using the cc-pVDZ and jun-cc-pVDZ Dunning correlation-consistent basis sets.^73−76^ The basis set jun-cc-pVDZ consists of diffuse functions added to all heavy atoms, except for the basis functions with the highest angular momentum. For the THC-RR-CCSD(T) computations, we set the eigenvalue tolerance of the MP2 projector to be 10^–4^. In other words, the ranks (*n*_*proj*_) of the doubles and triples projectors are determined from how many eigenvalues of the MP2 *t*_*ij*_^*ab*^ amplitudes are greater than 10^–4^, defined as τ from eq 29 in our work. For these computations, *n*_*proj*_ is around 400, compared to the max possible rank of 2205 (*n*_*occ*_*n*_*virt*_) in the cc-pVDZ basis, yielding a compression ratio of around 18%. Similarly, in the jun-cc-pVDZ basis, the ratio is 440/2793, which is around 16%.

**Figure 1:**
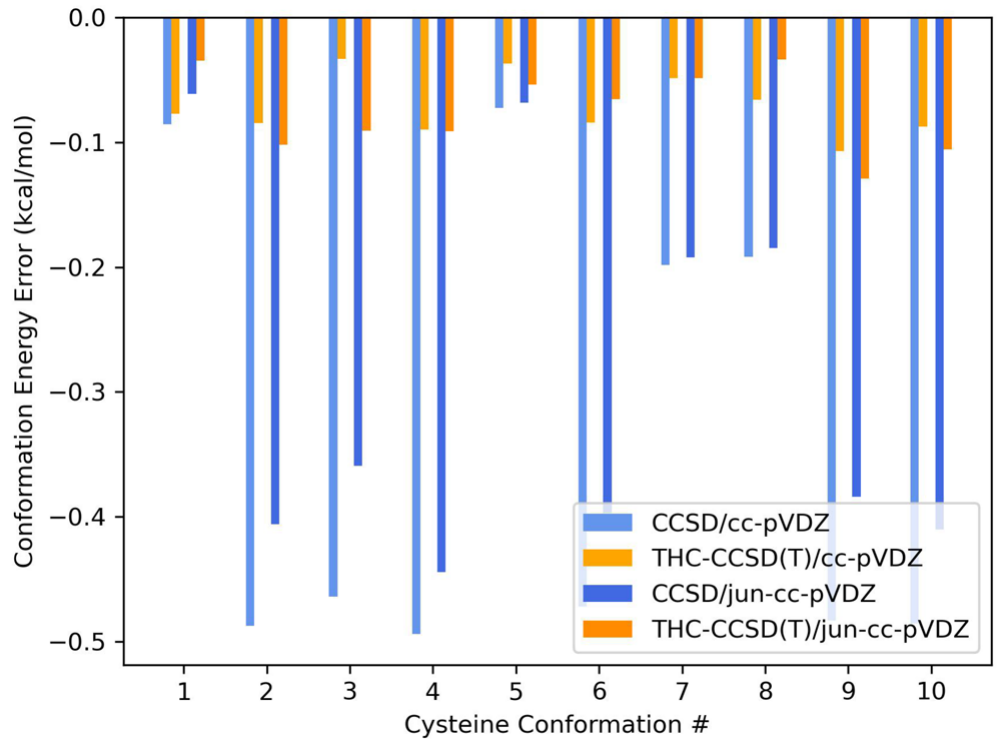
Errors in conformation energies for CCSD and THC-RR-CCSD(T) evaluated on the CYCONF data set, compared to the exact CCSD(T) reference, evaluated in with the cc-pVDZ and jun-cc-pVDZ basis sets, with a 10^–4^ eigenvalue tolerance.

The summary statistics are presented in Table 1, and the results for each individual conformation are presented in Figure 1. In the table, for the THC-RR-CCSD(T) algorithms, the eigenvalue tolerance is given in parentheses. To summarize the findings, THC-RR-CCSD(T) consistently gives lower errors compared to CCSD, for both basis sets, and the errors are on the order of less than 0.1 kcal/mol. The error also does not significantly grow with the addition of diffuse functions, from cc-pVDZ to jun-cc-pVDZ. The (T) correlation energy for these conformers is around 35–36 millihartrees for cc-pVDZ and 37–38 millihartrees for jun-cc-pVDZ. For both basis sets, THC-RR-CCSD(T) recovers around 98.5% of the (T) correlation energy for each conformer. It is further encouraging to note that the absolute energy errors for these sets of computations hover around 0.3–0.4 kcal/mol, such that the evaluation of relative energies benefits from favorable error cancellation. Detailed numbers for how much correlation energy is recovered for each conformer in each basis set are available in the Supporting Information (SI).

**Table 1:** Errors in Conformation Energy Compared to the Exact CCSD(T) Reference^a^

Test set	Mean error (kcal/mol)	MAE (kcal/mol)	RMSE (kcal/mol)	Std. dev. (kcal/mol)
CCSD/cc-pVDZ	–0.343	0.343	0.384	0.173
THC-RR-CCSD(T)/cc-pVDZ (10^–4^)	–0.072	0.072	0.075	0.023
CCSD/jun-cc-pVDZ	–0.291	0.291	0.323	0.141
THC-RR-CCSD(T)/jun-cc-pVDZ (10^–4^)	–0.076	0.076	0.082	0.031

a The number in parenthesis is the eigenvalue tolerance used to determine projector rank.

### 5.2 Potential Energy Surface

**Figure 2:**
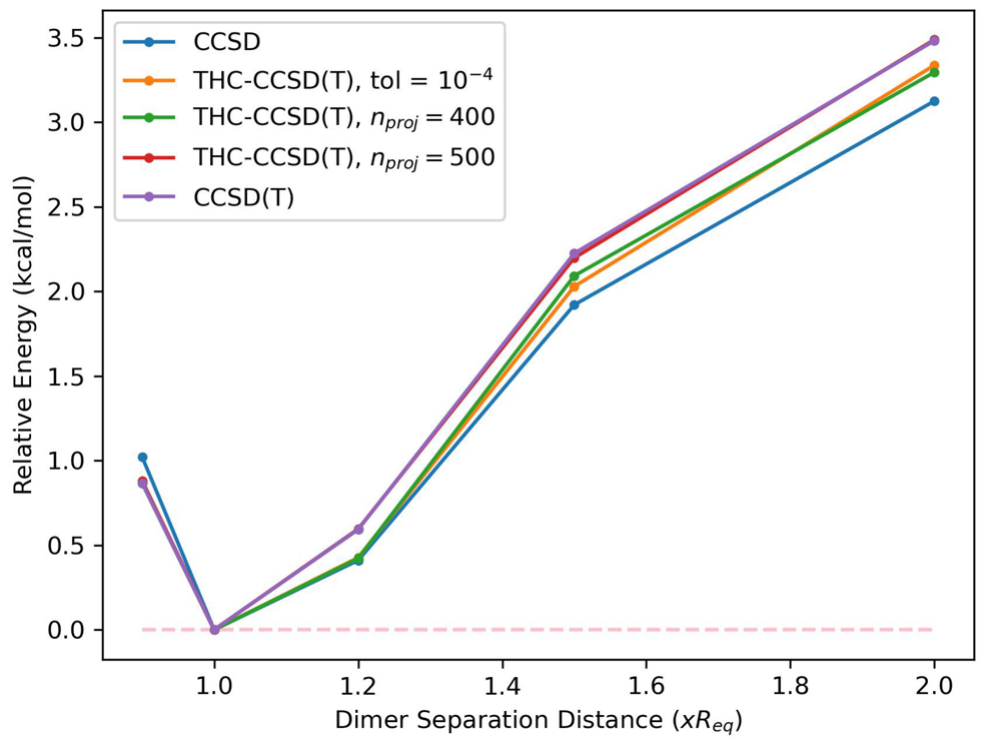
Relative energies of benzene–HCN dimer (S22 system 19) evaluated with each method at five different dimer separation distances relative to the equilibrium geometry.

We perform next, a potential energy surface scan on the benzene–HCN dimer system (compound 19 from the on S22 data set^77^), with the hydrogen atom of HCN pointing toward the π-bonds in the benzene. We measured the energy of the system at five different interatomic distances, relative to the equilibrium geometry, ranging from 0.9 to 2.0 times the equilibrium geometry length, with the geometries coming from the S22x5 data set.^78^ In Figure 2, we plot the shape of the potential energy surface of the THC-RR-CCSD(T) method at an eigenvalue tolerance of 10^–4^, as well as using predetermined projector ranks of 400 and 500. For all systems, an eigenvalue tolerance of 10^–4^ corresponds to a projector rank between 420 and 430. All THC-RR-CCSD(T) computations better capture the potential energy surface than the reference CCSD computations, with the computations with the predetermined projector ranks better capturing the shape of the surface than the one with a set eigenvalue tolerance. The THC-RR-CCSD(T) potential energy surface with *n*_*proj*_ set to 500 exactly matches the CCSD(T) potential energy surface, for practical purposes, with a max error of 0.027 kcal/mol and a RMSE of 0.014 kcal/mol. The shape of the potential energy surface, for each method, is shown in Figure 2, while the error statistics are presented in Table 2. For these systems, the magnitude of the (T) energy hovers around 48–49 millihartrees, and THC-RR-CCSD(T) recovers around 98%–99% of the correlation energy (around 0.3–0.6 kcal/mol) for all computations. The case with *n*_*proj*_ = 500 benefits from a more consistent percentage of the (T) correlation energy captured across the potential energy surface. Even though the absolute energy errors did not drop significantly, the relative energy errors did. Detailed numbers are available in the SI.

**Table 2:** Errors in Relative Energies Compared to the Exact CCSD(T) Reference, for a Reference CCSD Computation and THC-RR-CCSD(T) Computations with Varying Parameters

Test set	Mean error (kcal/mol)	MAE (kcal/mol)	RMSE (kcal/mol)	Std. dev. (kcal/mol)
CCSD	–0.138	0.200	0.236	0.191
THC-RR-CCSD(T), tol = 10^–4^	–0.100	0.103	0.132	0.086
THC-RR-CCSD(T), *n*_*proj*_ = 400	–0.098	0.098	0.128	0.082
THC-RR-CCSD(T), *n*_*proj*_ = 500	–0.001	0.010	0.014	0.014

### 5.3 Rank Convergence

**Figure 3:**
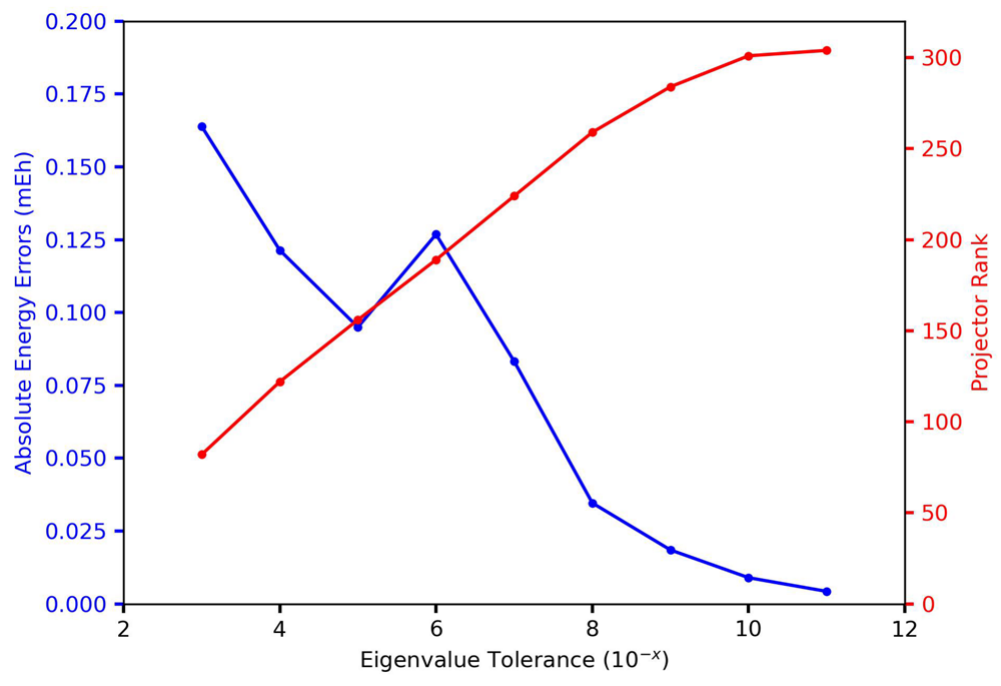
Convergence of the absolute energy of a water dimer system (S22), with respect to eigenvalue tolerance and rank.

Next, to demonstrate the convergence of the THC-RR-CCSD(T) method, compared to the exact CCSD(T) energy, we ran a series of computations of the water dimer from the S22 set,^77^ at eigenvalue tolerances from 10^–3^ to 10^–11^. An eigenvalue tolerance of 10^–11^ corresponds to no rank compression for this system. The errors with respect to eigenvalue tolerance and compression ranks are plotted in Figure 3, and it is encouraging to see the errors decrease smoothly to the true CCSD(T) energy, within the DF/RI approximation of the ERIs. The errors are on the order from 0.0 to 0.2 millihartrees, compared to the total (T) correlation energy of around 6.4 millihartrees. We attribute the “kink” in the graph from 10^–4^ to 10^–6^ as an artifact of the CP decomposition of the triples projector, with the CP error increasing slightly between the projector ranks of 122–156, before going back down. This artifact is well known on studies of the CP decomposition algorithm,^51^ where medium CP decomposition ranks suffer larger losses in accuracy compared to small or large ranks. Further studies and work are encouraged to look for ways to mitigate this phenomenon in the context of decomposing CC amplitudes.

### 5.4 Timings

**Figure 4:**
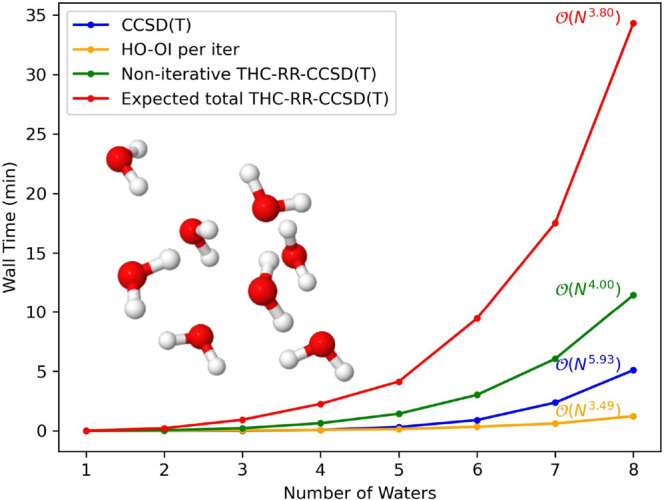
Timings, as well as scalings, for a growing system of water clusters in the cc-pVDZ basis set.

**Figure 5:**
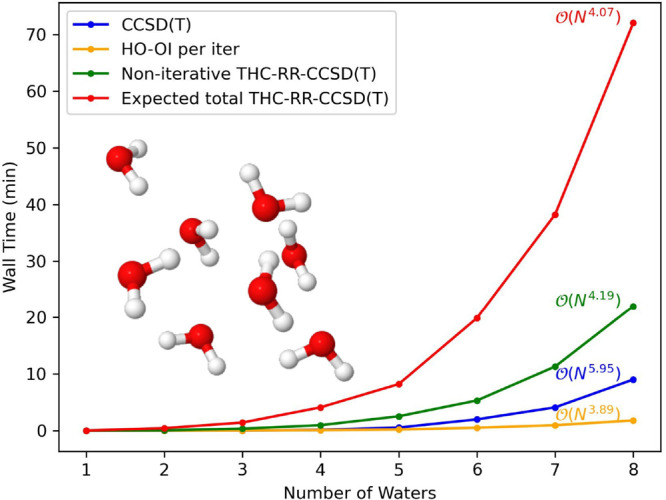
Timings, as well as scalings, for a growing system of water clusters in the jun-cc-pVDZ basis set.

**Figure 6:**
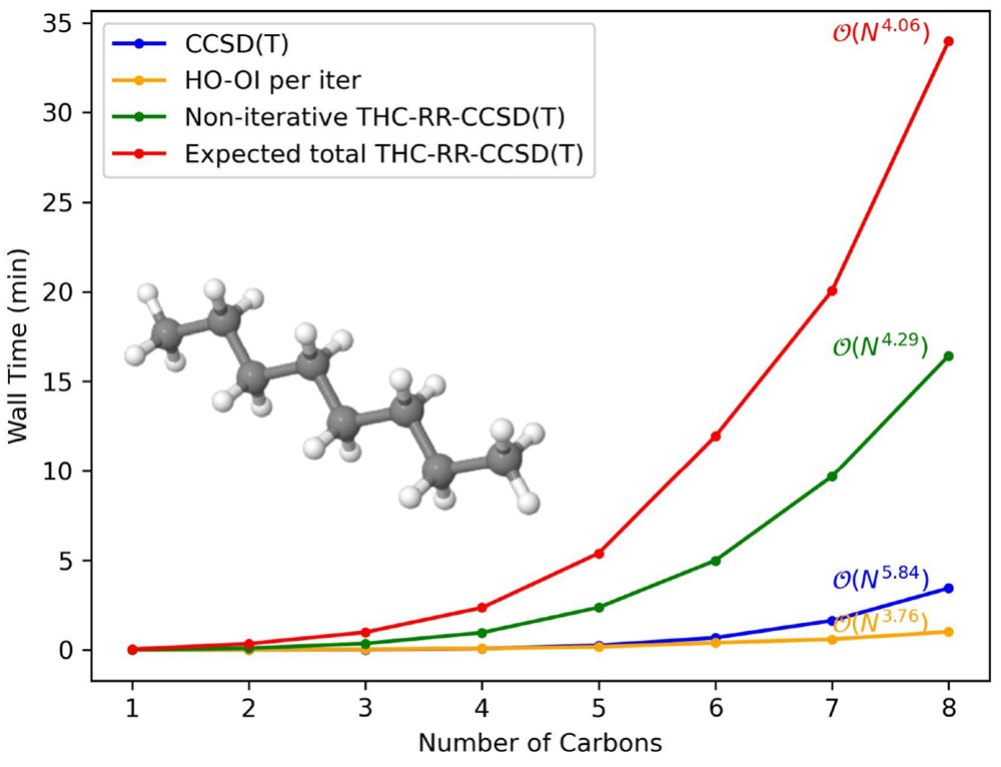
Timings, as well as scalings, for a growing system of linear alkanes in the cc-pVDZ basis set.

**Figure 7:**
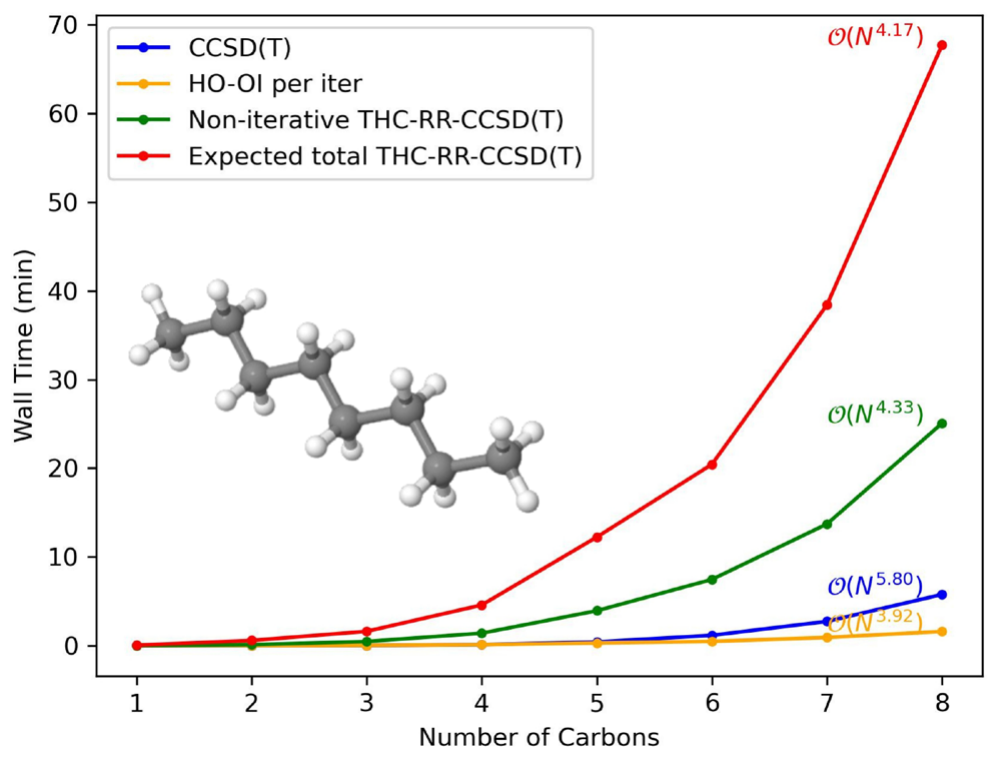
Timings, as well as scalings, for a growing system of linear alkanes in jun-cc-pVDZ basis set.

To establish the lower scaling of the THC-RR-CCSD(T) method compared to CCSD or CCSD(T), we present timings on growing systems of water clusters and linear alkanes, from 1 to 8 heavy atoms, in the cc-pVDZ and jun-cc-pVDZ basis sets. All computations are performed on 48 CPU cores of an Intel Xeon 6136. For each system and basis set combination, we present timings for THC-RR-CCSD(T) at a constant eigenvalue tolerance (10^–4^). In Figures 4–7, we present raw timings for the computation of the (T) energy and the noniterative steps in computing the THC-RR-(T) energy, as well as the raw timings for each iteration of the HO-OI procedure used in forming the triples amplitude projector. Since each computation requires a different number of iterations for convergence in the HO-OI procedure, instead of presenting raw timings for THC-RR-CCSD(T), we instead present an “expected time” for THC-RR-CCSD(T), which is the sum of the time required for the noniterative portion of the THC-RR-(T) computation, added with the average number of HO-OI iterations multiplied by the per iteration HO-OI time. A different average is computed across every system and basis set combination.

This “expected” time is also what is used when computing the scaling of the THC-RR-CCSD(T) method, as well as the crossover points with CCSD(T) in Tables 3 and 4. To compute the scaling of each method, we applied a power fit of the run time of each procedure in the form of *t* = *a* · *n*^*b*^, where *t* is the run time, *a* the prefactor, *n* the number of basis functions in the molecule, and *b* the computational scaling. The coefficients *a* and *b* are determined through a linear regression of log(*t*) as the independent variable and log(*n*) as the dependent variable. In our analysis, we only consider timings from systems with three or more heavy atoms, and the *r*^2^ coefficient of the linear regression is greater than 0.99 in all cases, showing the values shown in Tables 3 and 4 to be reliable. In all system and basis set combinations, computing the THC-RR-(T) energy scaled significantly better than computing the (T) energy or the preceding CCSD computation. The values are consistent with theoretical considerations, with the computation of the (T) energy scaling empirically around *O*(*N*^6^), and the computation of the THC-RR-(T) energy scaling around . This is consistent with there being few  steps in CCSD(T) and few  steps in THC-RR-CCSD(T). We note that though our current pilot implementation is not optimized, the crossover points presented for both systems are still reasonable and can be made much lower in future, more optimized implementations. Detailed numbers for timings, prefactors, scalings, and crossover points for each system tested are available in the SI.

**Table 3:** Empirical Scalings of Each Method for Water Clusters Taken with Respect to the Number of Basis Functions and Crossovers between CCSD(T) and THC-RR-CCSD(T)

Method	Scaling (cc-pVDZ)	Scaling (jun-cc-pVDZ)
CCSD	4.60	4.56
CCSD(T)	5.93	5.95
THC-RR-CCSD(T)	3.80	4.07
Crossover (basis functions)	319	418
Crossover (heavy atoms)	14	15

**Table 4:** Empirical Scalings and Crossovers of Each Method for Linear Alkanes and Crossovers between CCSD(T) and THC-RR-CCSD(T)

Method	Scaling (cc-pVDZ)	Scaling (jun-cc-pVDZ)
CCSD	4.56	4.51
CCSD(T)	5.84	5.80
THC-RR-CCSD(T)	4.06	4.17
Crossover (basis functions)	636	744
Crossover (heavy atoms)	27	27

### 5.5 Error Growth

**Figure 8:**
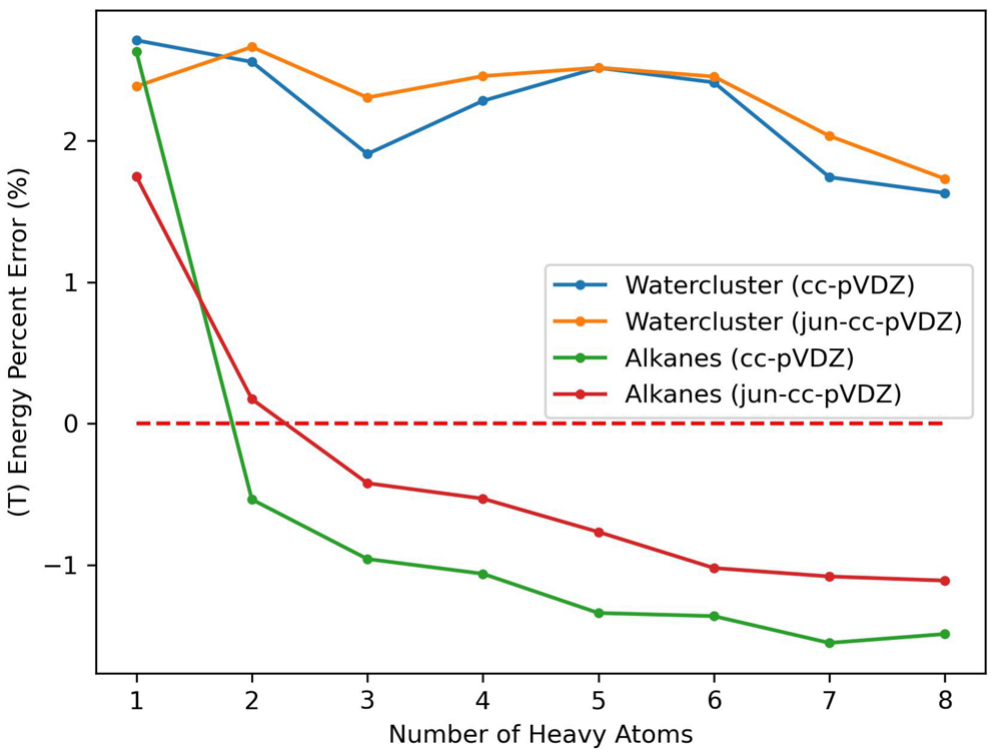
Percent errors of the (T) correlation energy for THC-RR-CCSD(T) evaluated with each system and basis set combination.

Finally, we plot the percentage errors of the (T) correlation energy for each combination of system and basis set used for timings in the previous section and show that the errors do not grow with larger systems, remaining under 3% in all cases (Figure 8). This shows the size extensivity of the THC-RR-CCSD(T) energy.

## 6 Conclusions

In this paper, we present the working equations for the THC-RR-CCSD(T) method, an  scaling approximation to CCSD(T), that allows for systematic control of errors. In our pilot implementation, we show the errors are controllable to the point of maintaining chemical accuracy of less than 0.1 kcal/mol for relative energies, and 1 mEh for absolute energies, while maintaining size extensivity. We also show that the method yields continuous potential energy surfaces that closely match the CCSD(T) surfaces with sufficient projector rank. In addition, we have empirically established that THC-RR-CCSD(T) indeed scales better than CCSD or CCSD(T). In the future, we hope to consider ways to improve the errors of the method at a given eigenvalue tolerance, such as through using other sources for the orthogonal projector. We would also like to look into alternative approaches to the THC factorization of orthogonal projectors. Though a CP decomposition is generally applicable, and relatively easy to implement, it does not assume any underlying form about the amplitudes. One avenue is the extension of the quadrature-based approach of Parrish, Hohenstein, Martínez, and Sherrill with Least-Squares Tensor Hypercontraction (LS-THC) to the triples amplitudes.^79,80^ Finally, we hope to have a more optimized implementation of this method available in the future, one that yields lower crossover points relative to CCSD(T), and available in the Psi4 package.^70^

## Data Availability

The data that support the findings of this study are available with the article and the Supporting Information.

## Supporting Information Available

Data that support the findings of this study (ZIP)
